# Innovations in the prevention and treatment of postpartum hemorrhage: Analysis of a novel medicines development pipeline database

**DOI:** 10.1002/ijgo.14200

**Published:** 2022-06-28

**Authors:** Annie R.A. McDougall, Maya Goldstein, Andrew Tuttle, Anne Ammerdorffer, Sara Rushwan, Roxanne Hastie, A. Metin Gülmezoglu, Joshua P. Vogel

**Affiliations:** ^1^ Maternal, Child and Adolescent Health Program Burnet Institute Melbourne Australia; ^2^ Policy Cures Research Sydney Australia; ^3^ Concept Foundation Geneva Switzerland; ^4^ Department of Obstetrics and Gynecology University of Melbourne Heidelberg Australia; ^5^ School of Public Health and Preventive Medicine Monash University Melbourne Australia

**Keywords:** innovation, medicines, postpartum hemorrhage, prevention, treatment

## Abstract

**Background:**

A significant barrier to improving prevention and treatment of postpartum hemorrhage (PPH) is a lack of innovative medicines that meet the needs of women and providers, particularly those in low‐and middle‐income countries (LMICs). The Accelerating Innovation for Mothers (AIM) project established a new database of candidate medicines under development for five pregnancy‐related conditions between 2000 and 2021.

**Objective:**

To systematically identify and rank candidates for prevention and treatment of PPH.

**Search Strategy:**

Adis Insight, Pharmaprojects, WHO ICTRP, PubMed, and grant databases were searched to develop the AIM database.

**Selection Criteria:**

AIM database was searched for candidates being evaluated for PPH prevention and treatment, regardless of phase.

**Data Collection and Analysis:**

Candidates were ranked as high, medium, or low potential based on prespecified criteria. Analysis was primarily descriptive, describing candidates and development potential.

**Main Results:**

Of the 444 unique candidates, only 39 pertained to PPH. One was high potential (heat‐stable/inhaled oxytocin) and three were medium potential (melatonin, vasopressin and dofetilide via nanoparticle delivery).

**Conclusion:**

The pipeline for new PPH medicines is concerningly limited, lacking diversity, and showing little evidence of novel technologies. Without significant investment in early‐phase research, it is unlikely that new products will emerge.

## Synopsis

A new database of maternal medicine development shows few new PPH candidates are in the pipeline. Significant investment is needed, particularly in early‐phase research.

## 1 INTRODUCTION

Postpartum hemorrhage (PPH) is commonly defined as maternal blood loss of 500 ml or more within 24 h of birth and affects approximately 6% of women giving birth.^1^, ^2^ Without treatment, postpartum hemorrhage can rapidly worsen and lead to hypovolemic shock, organ failure, and death.^3^ Obstetric hemorrhage is the leading cause of maternal death worldwide, causing 27% of the 295 000 maternal deaths occurring each year; more than two‐thirds of these are due to PPH.^4^, ^5^ The burden of PPH is significantly greater in low‐ and middle‐income country (LMIC) settings, and one‐third of all PPH‐related maternal deaths occurring globally take place in Asian and African countries.^4^, ^5^ The World Health Organization (WHO) recommends the use of an effective uterotonic—such as oxytocin, carbetocin, misoprostol, or ergometrine/methylergometrine—in the immediate postpartum period for all women giving birth to prevent PPH.^6^, ^7^ For women who experience PPH, first‐line management includes fluid resuscitation, additional uterotonics, tranexamic acid, and uterine massage, with additional more invasive interventions required if bleeding continues.^2^

Despite these effective measures, PPH remains a leading cause of maternal mortality and severe morbidity globally. A major contributing factor is a lack of access to good‐quality uterotonics in many health services worldwide.^4^ Studies have identified significant quality issues with oxytocin and other uterotonics in many LMICs.^8^ While oxytocin is the preferred first‐line uterotonic for PPH prevention and treatment, it requires cold‐chain transport and storage, as well as administration by an injection from a trained, skilled health worker—these requirements are not universally available in all settings where women give birth.^9^ As such, a significant barrier to reducing maternal mortality and severe morbidity due to PPH is a lack of innovative medicines that have been developed and evaluated for this indication, particularly those designed to meet the needs of women and providers in limited‐resource settings. This is reflective of a broad underinvestment in pharmaceutical research and development (R&D) of medicines for pregnancy‐specific conditions.^10^, ^11^ A 2008 analysis by Fisk and Atun^10^ identified only 67 drugs under development for any maternal health condition for the period 1980–2007, with only 17 in active development as of November 2007—only nine of which were new compounds. Comparatively, over the same period of time, 660 drugs were under development for cardiovascular indications and 34 drugs were being developed for amyotrophic lateral sclerosis, a comparatively rare neurodegenerative condition.

In the intervening years, the field of maternal health medicines development has continued to be stunted by a range of clinical, ethical, financial, and legal barriers. That said, general advancements in clinical research and medicines development during this period, as well as the growth of innovative push‐pull mechanisms—including an upspring of novel product development partnerships for neglected diseases and conditions—has fueled motivation to build on Fisk and Atun’s important research and provide an updated landscape analysis of maternal health medicines R&D.

The Accelerating Innovation for Mothers (AIM) project was established in 2020 to foster greater investment in and development of critical maternal health medicines for five pregnancy‐related conditions where biomedical product gaps exist (preterm labor/birth, pre‐eclampsia/eclampsia, intrauterine growth restriction, PPH, and intrapartum fetal distress). A significant part of AIM involved the development of a database of candidates for these potential maternal health conditions over the period 2000–2021.^12^ In the present paper, we analyzed candidates within the AIM database specific to prevention and treatment of PPH and categorized those that are most promising in terms of reducing PPH‐related maternal morbidity and mortality globally.

## 2 MATERIALS AND METHODS

### 2.1 Development of a medicines pipeline database

We established a comprehensive database of candidates for preventive or therapeutic medicines (drugs, biologics, and dietary supplements) that have been investigated either clinically or preclinically between the years 2000 and 2021. The complete methodology for the development of this database is described in detail in Supporting Information S1, including web addresses for databases used in the search. In brief, the overall strategy was to undertake a series of partially sequential, partly overlapping steps to develop a database of candidate profiles. As this was an analysis of available literature, ethics approval was not required nor sought.

**FIGURE 1:**
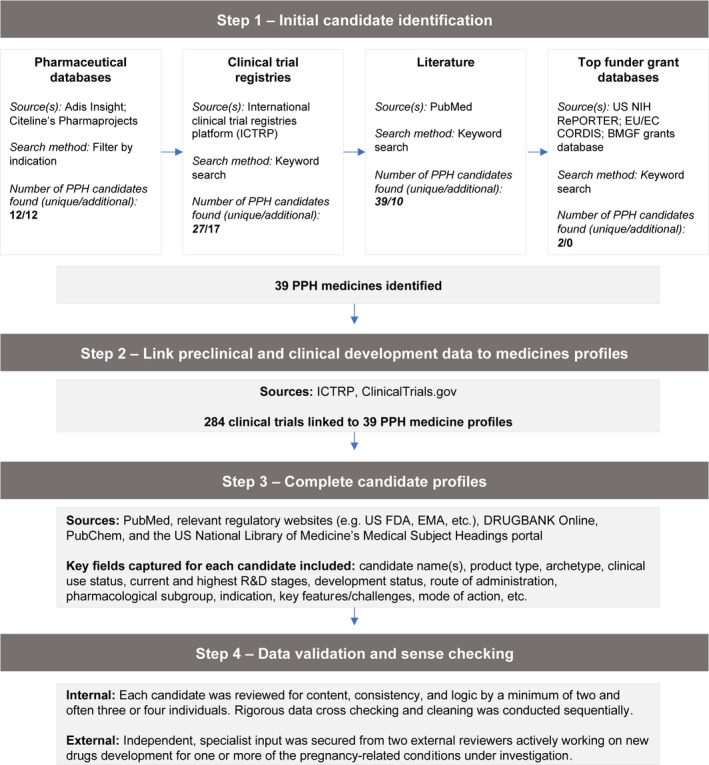
Overview of development of Accelerating Innovation for Mothers (AIM) medicines pipeline database

First, we identified and validated candidates through multiple databases and sources. Targeted searches of multiple drug development databases were screened and merged to create a single database of candidate profiles for the five conditions of interest (Figure 1). The database summarizes information on each candidate’s preclinical or clinical development as well as relevant contextual information (e.g. development history, key stakeholders, etc). Fields of interest included candidate name(s), pregnancy‐specific condition, product type (drug, biologic, dietary supplement), archetype (repurposed, new chemical/biological entity), clinical use status for this pregnancy condition (approved, used off‐label, etc), current R&D stage, highest R&D stage, development status (active, inactive), route of administration, pharmacological subgroup, indication, key features/challenges, mode of action, and other indications investigated.

Adis Insight—a leading drug development database with more than 200 000 drug, trial, deal, and safety records—was searched in January 2021. We then cross‐checked identified candidates with Citeline’s Pharmaprojects database—a subscription‐only global pharma R&D database with over 68 000 drug profiles for over 15 000 candidates—to both validate identified and uncover additional candidates. Next, we searched the WHO International Clinical Trials Registry Platform (ICTRP) in January 2021. Clinical trials were scoped for relevance, which we defined as an investigation of one or more drugs, biologics, or dietary supplements with a primary and/or secondary outcome measure matching at least one of the five conditions. This search served to uncover additional candidates for inclusion that had not yet been identified, as well as to capture and link clinical trial data to included candidates (see below). We also searched the grant databases of three of the largest global funders of medicines development to validate existing and find new candidates (particularly those in preclinical/discovery stage): the United States National Institutes of Health (US NIH)’s RePORTER; the European Union/Commission’s CORDIS; and the Bill & Melinda Gates Foundation (BMGF) grants database (supplied from BMGF). For RePORTER, we retrieved all grants dating from 2000 to present. For CORDIS, we retrieved four datasets relating to different EC program cycles: FP5 1998–2002; FP6 2002–2006; FP7 2007–2013; and Horizon 2014–2020. For BMGF, we searched datasets ranging from 2014–2019 inclusive. All datasets were retrieved and scoped for relevance between March and April 2021.

In addition, we searched PubMed for relevant literature to validate already identified and uncover new candidates for inclusion. PubMed searches were conducted between March and April 2021. Returned paper titles and abstracts were reviewed for relevance, with relevant publications reviewed in full. Based on information synthesized from aforementioned sources, individual profiles were generated for each candidate. The complete database of unique preventative and therapeutic candidates was assembled, with initial validation checks performed to identify and remove any duplicate entries or transcription errors.

Second, we collected information on the candidate’s preclinical or clinical development, and associated data. For candidates in clinical development, we collected relevant clinical trial data through already mentioned sources, i.e. Adis Insight and ICTRP. We further cross‐checked candidates against relevant national clinical trials registers where additional clinical trial information was required. For candidates in preclinical development, results were sourced through PubMed searches for published research findings.

Third, we researched additional context around the product (e.g. development history, stakeholders) to complete candidate profiles as much as possible. We conducted additional focused per‐candidate searches of PubMed and relevant regulatory websites, such as the US Food and Drug Administration and European Medicines Agency. Information for other database fields was sourced from reliable, public access databases, including DRUGBANK Online, PubChem, and the US National Library of Medicine’s Medical Subject Headings portal.

Fourth, we validated and sense‐checked candidate profiles through rigorous review. The database was internally validated by review of content, consistency, and logic by at least two (often three or four) individuals on our research team per candidate. The entire database was then reviewed externally by two independent specialist reviewers actively working on drug development for one or more of the pregnancy‐related conditions under investigation. The database will be made freely available online via the AIM project website in late 2021.

### 2.2 Database analysis for PPH candidates

All candidates in the database can be filtered by type (drug, dietary supplement, or biologic), drug subclass, and clinical research phase (amongst others). Candidates designated as Phase I/II were incorporated into the Phase I category. For this analysis, we identified all unique candidates where prevention or treatment of PPH was specified in the candidate profile. Two authors independently reviewed each candidate and classified them using a prespecified flowchart (Figure 3). First, we excluded candidates that have been approved for PPH and are available on the market and/or are widely recommended or used off‐label. Remaining candidates were filtered by development status—either active or inactive. Inactive candidates were those that were discontinued or where no updates had been reported since 2019. Candidates that were inactive due to negative trial outcomes were excluded (such as adverse maternal or neonatal outcomes). The candidates left were then reviewed; candidates were excluded if they were either inferior to current available treatments or if they were already recommended to a subgroup of women experiencing PPH (i.e. women with clotting disorders).

The remaining included candidates were ranked as high, medium, or low potential, through a consensus process involving all authors, on the basis of prespecified criteria. Candidates were considered high potential if the results from preclinical or clinical trials were promising, with rigorously designed and reported studies, and the candidate had potential to reach LMIC markets. Medium potential was assigned if the candidate had promising results from preclinical or clinical trials but with large uncertainty, and the candidate had potential to reach LMIC markets. Low potential was assigned if the candidate had promising results from preclinical or clinical trials, but with large uncertainty and low potential for LMIC markets.

## 3 RESULTS

The AIM database identified 444 unique candidates across the five conditions. The conditions with the largest number of candidates were management of preterm labor/birth (178 candidates) and prevention and treatment of pre‐eclampsia (153 candidates). Candidates for prevention and treatment of PPH were comparatively limited, with 39 unique candidates identified (Figures 1).

**FIGURE 2:**
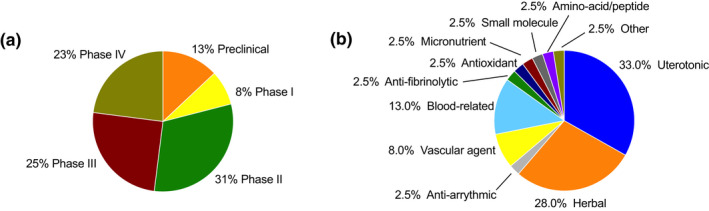
Research stage (a) and subclass of candidates (b) in the pipeline for prevention and treatment of postpartum hemorrhage (*n* = 39)

Of the 39 candidates, 27 (69.2%) were currently active and 12 (30.8%) were inactive. Five candidates were in preclinical stage (12.8%), three were in Phase I (7.7%), 12 were in Phase II (30.8%), 10 were in Phase III (25.6%), and 9 were in Phase IV (23.1%; Figure 2a). Twelve candidates were classified as dietary supplements (30.8%, mostly traditionally used herbs), five were biological (12.8%), and 22 were classified as drugs (56.4%). One‐third of all PPH medicines (13 candidates, 33.3%) were also new chemical/biological entities (NCEs), with the remaining repurposed medicines (26 candidates, 66.7%).

**FIGURE 3:**
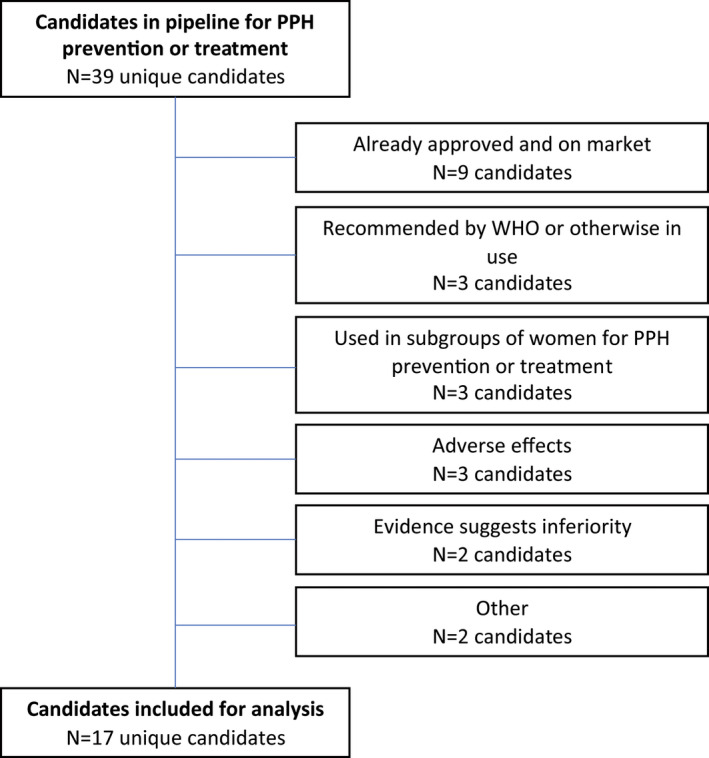
Flowchart for assessing eligibility of candidates in pipeline for prevention and treatment of postpartum hemorrhage (PPH)

After applying the decision tree (Figure 3), 22 candidates were excluded from further analysis. Nine were excluded as they were already approved and on the market (ergometrine/methylergometrine, oxytocin/ergometrine ‐ fixed dose combination, prothrombin complex concentrate [PCC], fresh frozen plasma [including cryoprecipitate], oxytocin, carbetocin, dinoprost, sulprostone, carboprost). A further three products are recommended by WHO or otherwise available and in use (tranexamic acid, misoprostol, carbetocin heat stable). Three candidates are already used for selected subgroups of women for PPH prevention or treatment (desmopressin, recombinant von Willebrand factor, eptacog alfa). Three candidates were inactive and excluded due to identified adverse effects in available research (Shenghua decoction, Phorbol‐12,13‐dibutyrate, dinoprostone [PGE2]); two were excluded as available evidence indicates they are inferior to other options (VU 590 dihydrochloride and heat‐stable sublingual disintegrating oxytocin); and one was excluded for endpoints not being met in a clinical trial (vitamin K). Oxytocin (Uniject injection device) was excluded as oxytocin is currently on the market, and the Uniject device is no longer in production.

Seventeen candidates remained for further analysis (Figure 2b). Of these, two were in the preclinical stage of development, two in Phase I, nine in Phase II, and four in Phase III. Candidates in the preclinical stage included an antiarrhythmic (dofetilide with nanoparticle delivery) and a beta‐blocker (propranolol). Candidates in Phase I included a uterotonic (heat‐stable inhaled oxytocin) and a blood‐related product (ethamsylate). Candidates in Phase II included a vascular agent (vasopressin), an antioxidant (melatonin), and seven herbal supplements (motherwort, grape seed extract, dill extract, *Urtica dioica* extract, *Plantago*, *Nigella sativa*, and *Anethum graveolens* extracts, and *Capsella bursa‐pastoris*). Candidates in Phase III included three herbal supplements (*Portulaca oleracea* extract, *Phoenix dactylifera* extract, and *Achillea millefolium* extract) and a blood‐related factor (fibrinogen concentrate). Following ranking of the 17 candidates, 1 was high potential, 3 were medium potential, and 13 were low potential.

### 3.1 High‐potential candidates

Only one was ranked as high potential: heat‐stable/inhaled oxytocin (Table 1). Oxytocin is an established first‐line medicine for PPH prevention and treatment.^6^ Preclinical models have demonstrated pharmacokinetics of heat‐stable/inhaled delivery is comparable to injectable delivery.^13^ The heat‐stable inhaled oxytocin is currently in Phase I. It has high potential in LMIC markets as it removes the need for cold‐chain storage and can facilitate administration in those settings where injectable oxytocin is not consistently available.

**TABLE 1:** Characteristics of candidates for prevention and treatment of postpartum hemorrhage, ranked as high or medium potential

Name	Product type	Archetype	R&D stage for PPH	Indication	Administration route	Drug subclass
High‐potential candidates						
Oxytocin – heat stable and inhaled	Drug	New chemical or biological entity	Phase I	Prevention and treatment of PPH	Inhalation	Uterotonic
Medium‐potential candidates						
Melatonin	Drug	Repurposed	Phase II	Prevention and treatment of PPH	Sublingual, oral	Antioxidant
Vasopressin	Drug	Repurposed	Phase II	Prevention and treatment of PPH	Intravenous, intramyometrial, subcutaneous	Vascular agent
Dofetilide (nanoparticle delivery)	Drug	New chemical or biological entity	Preclinical	Prevention and treatment of PPH	Oral	Antiarrhythmic

### 3.2 Medium‐potential candidates

Melatonin, vasopressin, and the antiarrhythmic drug dofetilide (nanoparticle delivery) were ranked as medium potential (Table 1). Melatonin is a hormone involved in regulating the sleep–wake cycle. Melatonin levels peak in pregnant women just before birth, and as such have been posited to signal increased myometrial contractility, like oxytocin.^14^, ^15^ In vitro studies show a promising role for melatonin in promoting increased myometrial contractility by synergizing with oxytocin,^16^ and a clinical trial of 120 women undergoing cesarean section in India suggested melatonin might reduce blood loss.^17^ Its use following vaginal delivery remains unclear; a trial registered in 2015 has not been subsequently updated (Iranian Registry of Clinical Trials [IRCT] 2015050919037N9).

Vasopressin is a hormone that is structurally similar to oxytocin. It is used for several clinical conditions, including variceal bleeding, diabetes insipidus, and vasodilatory shock. Given the potent vasoconstriction actions of vasopressin and evidence of its effectiveness in treating PPH in a case series of 24 women,^18^ vasopressin is now being tested in clinical trials to control postpartum bleeding in high‐risk women including: after cesarean in women with placenta previa (NCT03725553), following postpartum hysterectomy, and in combination with balloon occlusion in women with placenta accreta (NCT03273569).^19^, ^20^

Dofetilide is a class III antiarrhythmic agent that prolongs the action potentials and promotes uterine contraction through inhibition of hERG channels.^21^ It is used for the maintenance of sinus rhythm in individuals prone to the occurrence of atrial fibrillation and flutter arrhythmias, and for chemical cardioversion to sinus rhythm from atrial fibrillation and flutter.^21^ Nanoparticle delivery is novel in the PPH context; nanoparticle delivery systems have been designed to deliver therapeutic agents to a target site or organ in a controlled manner, maximizing efficacy while minimizing off‐target effects. Liposomes loaded with dofetilide targeting the oxytocin receptor have been shown to increase contraction duration in myometrial tissue in vitro.^22^ Further preclinical animal studies and clinical trials will be required to assess efficacy and safety in pregnant and lactating women. While promising, nanoparticle delivery systems are not yet in common use for any indication in pregnant women.^23^

### 3.3 Low‐potential candidates

Thirteen candidates were ranked as low potential (Table 2). These included 10 herbal supplements (grape seed extract, dill extract, motherwort, *Urtica dioica* extract, *Portulaca* extract, *Plantago* extract, *Phoenix dactylifera* fruit, *Nigella sativa* and *Anethum graveolens* extracts, *Capsella bursa*‐*pastoris* and *Achillea millefolium* extract), two blood‐related products (fibrinogen concentrate and ethamsylate), and propranolol.

**TABLE 2:** Characteristics of candidates for prevention and treatment of postpartum hemorrhage, ranked as low potential

Name	Product type	Archetype	R&D stage	Administration route	Drug subclass
Low‐potential candidates					
Grape seed extract	Dietary supplement	Repurposed	Phase II	Oral	Herbal
Dill extract	Dietary supplement	Repurposed	Phase II	Oral	Herbal
Motherwort	Dietary supplement	Repurposed	Phase II	Intravenous, intramuscular	Herbal
*Urtica dioica* extract	Dietary supplement	Repurposed	Phase II	Oral	Herbal
*Portulaca* extract	Dietary supplement	Repurposed	Phase II	Oral	Herbal
*Plantago* extract	Dietary supplement	Repurposed	Phase II	Oral, rectal, topical	Herbal
*Phoenix dactylifera* fruit	Dietary supplement	Repurposed	Phase III	Oral	Herbal
*Nigella sativa* and *Anethum graveolens* extracts	Dietary supplement	Repurposed	Phase II	Oral	Herbal
*Capsella bursa‐pastoris*	Dietary supplement	Repurposed	Phase II	Oral	Herbal
*Achillea millefolium* extract	Dietary supplement	Repurposed	Phase III	Oral	Herbal
Fibrinogen concentrate	Biologic	Repurposed	Phase III	Intravenous	Blood related
Ethamsylate	Drug	Repurposed	Phase I/II	Intravenous	Blood related
Propranolol	Drug	Repurposed	Preclinical	Intravenous, oral	Beta blocker

The identified herbal supplements have limited evidence of effectiveness. While these supplements are often used in traditional medicines to reduce bleeding, current trials are ongoing and have not reported any evidence demonstrating effectiveness of *Urtica dioica (*IRCT 20160112025983N3*)*, *Portulaca* extract (IRCT 20181007041266N1), *Nigella sativa* and *Anethum graveolens* extracts (IRCT 20200214046492N1), or *Achillea millefolium* extract (IRCT 20160112025983N2). There is some trial evidence that motherwort, grape seed extract, dill extract, *Capsella bursa‐pastoris*, date fruit (*Phoenix dactylifera* fruit) and *Plantago* extract may be effective in preventing PPH; however, identified studies were generally low quality with small sample sizes.^24^, ^25^, ^26^, ^27^, ^28^, ^29^ The biological plausibility of supplements is uncertain, with the active compounds responsible for their effect or mechanism of action yet to be determined.

Fibrinogen (factor I) is an endogenous glycoprotein that circulates in the blood and is involved in blood clotting.^30^ Fibrinogen concentrate is used in patients with congenital fibrinogen deficiency to treat acute bleeding.^30^ Observational studies have demonstrated that low fibrinogen levels in the early stage of PPH are associated with increased blood loss and need for blood transfusion, suggesting that fibrinogen concentrate may be effective in treating PPH.^31^, ^32^, ^33^ A systematic review found that there is insufficient evidence that fibrinogen is effective in treating PPH and does not reduce the need for blood transfusion or improve outcomes associated with PPH.^34^ Ethamsylate is a hemostatic agent used to control bleeding from small vessels and to prevent neonatal intraventricular hemorrhage.^35^ There is limited evidence for ethamsylate in the PPH context: two registered trials have yet to report results (NCT02604719, NCT04656067). Propranolol is a beta blocker that may assist with re‐sensitizing the oxytocin‐desensitized myometrium, thus improving myometrial contractions. A recent trial comparing time to delivery in women undergoing induced labor treated with either propranolol or placebo found no significant difference in incidence of PPH, but did show a reduction in the need for blood transfusion following propranolol.^36^ As a nonselective beta blocker, propranolol’s possible adverse effects need to be carefully considered.

## 4 DISCUSSION

We systematically analyzed the R&D pipeline for research on novel or repurposed medicines to prevent or treat PPH over the past 20 years, identifying only 39 candidates. Currently, nearly 40% of these candidates are already marketed, widely used off‐label, or are recommended for a subgroup of women who are at increased risk of PPH. Only 10% of candidates in the development pipeline were ranked as high or medium potential for PPH prevention or treatment.

Several issues were highlighted by our analysis. Firstly, in addition to the small number of candidates overall, there is a lack of candidates at the early stages of development, particularly at preclinical and Phase I trial stage. Across the 39 candidates, only five were in the preclinical stage, compared to 19 in Phases III or IV. However, of the 10 candidates in Phase III, over half are already used for PPH, and the nine candidates in Phase IV are already approved. Taken together, this strongly suggests a decline in novel research activity over time, as opposed to the advancement of a large number of new candidates for this condition. Clinical trials of new candidates for PPH prevention or management face an additional challenge, given the technical difficulties in reliably measuring blood loss after birth, and the variable definitions of PPH.^37^ Given the long lag time between preclinical evaluation and translation to clinical practice, it also means there is likely to be few or zero new PPH medicines reaching the market in the foreseeable future. To rectify this, significantly greater investment in early phase R&D is required, and soon.

Second is the lack of diversity of candidates; almost two‐thirds were uterotonics or herbal supplements, although this probably reflects the existence of pre‐existing effective medicines for PPH.^6^ Many approved PPH medicines—while highly effective—are suboptimal for use in a variety of contexts, particularly LMICs. This is perhaps comparable to the contraceptive R&D landscape, where a reasonable number of highly effective options exist, but they are not always fit for purpose for all populations and settings, leaving much room (and need) for continued improvement or new products. The database did identify a handful of candidates specifically designed with LMICs in mind, including reformulations of effective uterotonics such as oxytocin in a subcutaneous Uniject device; sublingual, orally disintegrating oxytocin; inhalable, heat‐stable oxytocin; and heat‐stable carbetocin. A heat‐stable oxytocin that can be used for PPH prevention and treatment, as well as for labor induction and augmentation, would be—in our opinion—a useful solution to the well‐described challenges of oxytocin quality, transport, storage, and implementation. In this regard, the heat‐stable/inhaled oxytocin product that was classified as a high‐potential candidate is a promising R&D area.

In general, herbal supplements have some advantages: oral administration, potentially lower costs, wider availability, and they may be perceived (correctly or not) as having an acceptable safety profile in pregnancy. They may be viewed as natural supplements and not drugs, which could be more acceptable to women.^38^ There is however often a lack of regulation of supplements even if they have pharmacological properties. There is not yet compelling evidence for the effectiveness of herbal supplements for PPH prevention or treatment, and there is uncertainty about specific active compounds, biological mechanisms, and possible interactions with other medicines or supplements.^39^ Thus, their potential remains uncertain, and high‐quality clinical trials are required.

There were limited examples of novel therapies in the PPH candidate pipeline. While innovations around heat‐stable formulations and delivery mechanisms for uterotonics are novel, only two candidates not already approved for PPH were genuinely new entities—VU 590 dihydrochloride and Phorbol‐12,13‐dibutyrate—although VU 590 dihydrochloride was excluded in our analysis as preclinical data suggest it would not be useful as a uterotonic.^40^ The use of nanotechnology to directly target the uterus and deliver dofetilide (or other medicines) may be a promising technological advance^22^; significant advances have been made in oncology and cardiology with small molecules and nanomedicine.^41^, ^42^

We created a novel, robust database of medicines development for potential maternal health conditions since 2000, using a comprehensive, multipronged search strategy and several review and quality‐assurance steps to maximize coverage and accuracy. However, due to the proprietary nature of (and lack of publicly available information on) many candidates (particularly in preclinical stages), it is possible that some candidates were not identified, or information on those we did is incomplete. It is also possible that research groups may fail to report negative findings, particularly in preclinical stages, meaning that additional candidates may exist. We also acknowledge that some data sources we used rely on self‐reporting (e.g. ICTRP), which is subject to reporting bias. Available medicines were classified using prespecified criteria to identify high‐potential candidates for further investment, nevertheless we acknowledge that these assessments were based on available (or known) data on benefits and possible harms, as well as potential to reach LMIC markets. However, these assessments did not consider other factors affecting their use in clinical settings, such as women’s acceptability of the medicine, and their ease of use by providers. Lastly, medicines R&D is a dynamic field, and data presented here are up to date as of mid‐2021. The database was conceived as an ongoing, “living” database that will be regularly updated, and the potential of candidates will change to reflect advances in the field. For example, ongoing research investigating alternative routes of administration and formulations of tranexamic acid—such as the ongoing WOMAN‐PharmacoTXA trial—will likely warrant inclusion in future database updates.^43^

In conclusion, the research and development pipeline for new medicines to prevent and treat PPH is limited and lacking diversity. Much of the available research focuses on improved uterotonics and herbal supplements, with few promising preclinical candidates and little evidence of novel medicines. The most promising candidate identified was an innovative heat‐stable, inhaled oxytocin product in Phase I. Without significant, immediate investment in early‐phase research, it is unlikely that the range of available products to address PPH will broaden in the foreseeable future.

## CONFLICT OF INTEREST

AMG and AA are Concept Foundation staff who provide regulatory assistance to the developers of heat‐stable carbetocin and inhaled oxytocin. SR reports funding to Concept Foundation from MSD for Mothers. AA, AMcD, AMG, AT, JPV, and MG report grant funding to Concept Foundation from the Bill & Melinda Gates Foundation (Investment ID INV‐023749). Outside the present study, A. M. G. reports an honorarium from Ferring Pharmaceuticals paid to Concept Foundation for PPH panel participation (International Confederation of Midwives Conference, June 23, 2021). Other authors have no conflicts of interest to declare.

## AUTHOR CONTRIBUTIONS

The initial concept for this article was conceived by AMG, with the design developed by ARAM, MG, AT, AA, AMG, and JPV. Development of the database was conducted by MG and AT, with input from AA, SR, and AMG. Analysis of the database was conducted by ARAM, AA, RH, AMG, and JPV, with input from MG, AT, and SR. The first draft of the manuscript was written by ARAM and JPV, with MG, AT, AA, SR, RH, and AMG providing substantive input.

## Supporting information


Appendix S1
