# Supplementary material for: Innovations in the prevention and treatment of postpartum hemorrhage: Analysis of a novel medicines development pipeline database
Source: Int J Gynaecol Obstet. 2022 Jun 28;158(Suppl 1):31–9. doi: 10.1002/ijgo.14200 (PMC9328148; doi:10.1002/ijgo.14200)
Supplement: Supplementary file 1 — Appendix S1 [file IJGO-158-31-s001.docx]

Supporting information S1.

Methodology for medicines development for five pregnancy-related conditions (2000–2021)

Our approach was to create a comprehensive database profiling all medicines (drugs, biologics, and dietary supplements) investigated since 2000 for each pregnancy-related condition. Medicines could be applicable for use in any context, including high-income country (HIC) and low- and middle-income country (LMIC) contexts, or be targeted towards special subgroups, such as PPH prevention for women with Von Willebrand’s disease. For inclusion in the dataset, the medicine candidates needed to:

- be small molecules (drugs), biologics, or dietary supplements, with no restrictions: candidates could be entirely new entities; existing/repurposed/label extensions; new formulations or dosing of existing/registered medicines.
- have an indication or multiple indications related to the project’s five identified pregnancy-related conditions: preterm labor/birth, pre-eclampsia/ eclampsia, intrauterine growth restriction, postpartum hemorrhage, and fetal distress.
- either be in active development, or have been at one point between 2000 and 2020 (public announcements and updates on relevant candidates made between January and May 2021 were also captured in their respective profiles).
- be either investigated for clinical use and/or used currently in clinical treatment of the five identified conditions.

Specific exclusions were:

- devices, diagnostics, and other nonmedicine-related biomedical products with indications specific to the project’s five pregnancy-related conditions.

We undertook a series of partially sequential, partly overlapping, but mutually reinforcing steps to develop a database of candidate profiles. These were: (1) identify and validate candidates through multiple sources that are or were in the pipeline for each of the identified pregnancy-related conditions since 2000; (2) collect information on the candidate’s preclinical or clinical development, and associated data; (3) research additional context around the product (e.g. development history, stakeholders); and (4) validate and sense-check candidate profiles through independent, external reviews by clinical research specialists in the field.

Data requirements

These four research steps were borne out of an initial data requirement gathering exercise, whereby we agreed to and defined data fields to be captured for each candidate (where available and verifiable) (see Table 1).

Table 1. Data fields captured for each candidate (where available).

| CANDIDATE PROFILE | LINKED CLINICAL TRIAL (CT) DATA |
| --- | --- |
| - Candidate ID (internally assigned number) - Candidate name - Alternative/previous candidate names - Pregnancy-specific condition (primary) - Indication - Archetype - Product type - WHO ATC code - Medical subject headings - Pharmacological subgroup - Route of administration - Target - Mode of action - Clinical use status - Current R&D stage (for this pregnancy condition) - Highest R&D stage (for any condition) - Development status - Inactive development type (as appropriate) - Key features/challenges - Most recent update - FDA pregnancy labelling/pregnancy risk summary - Preclinical results status - Preclinical results type (as appropriate) - Preclinical results source (as appropriate) - Investigated for other indications (y/n) - Other indications (as appropriate) - Developer(s) - Patent - CAS number - Chemical name | - CT title - CT number - CT last updated - CT phase - CT source - CT status - CT terminated type (as appropriate) - CT terminated reason (as appropriate) - CT description - CT start date - CT start type (actual vs planned) - CT end date - CT end type (actual vs planned) - CT location(s) - CT enrolment - CT results status - CT results type (as appropriate) - CT results source (as appropriate) - CT sponsor(s) - CT collaborator(s) |

For each field, we developed a definition, data input description, and sample data type classification (for example, numeric, free-text, or defined list, etc), as well as guidance notes where relevant, to ensure standardized data entry across researchers/enumerators.

Methods and sources

Step 1: Initial candidate identification

Various sources were utilized to uncover and identify a total of 444 unique candidates.

(a) We searched Adis Insight^[[1]](#footnote-1)^ – a leading drug development database – to retrieve a comprehensive output of relevant pipeline drugs related to the project’s five pregnancy-related conditions. The platform returns detailed information on drugs, candidate deals, clinical trials, safety, patents, and other historical information useful for building candidate profiles. Information is full via subscription (our approach) or limited via open source. We searched utilizing Adis Insight’s inbuilt “by indication” function, which classifies drugs using a standardized list of indications. Accordingly, free-text searches by indication are not possible. We therefore used search terms that were the most relevant available indications in the database to the five conditions investigated. These were: "Preterm-labor" OR "Preterm labour" OR "Preterm birth" OR "Preterm-delivery"; "Pre-eclampsia" OR "Eclampsia"; "Foetal-growth-retardation" OR "Fetal growth retardation"; "Postpartum-bleeding" OR "Postpartum haemorrhage" OR "Postpartum-hemorrhage" OR "Delayed-postpartum-haemorrhage"; "Foetal-distress" OR "Fetal distress".

“Drug” outputs were retrieved and unique candidates and associated data formatted, extrapolated, and transposed into our database. Of note, postpartum hemorrhage and fetal distress returned zero “drugs” profiles but a number of associated “clinical trials” (39 and one respectively). For these conditions, we instead reviewed these linked clinical trials to identify unique candidates for inclusion in the database. Adis Insight search results were retrieved in January 2021 (see Table 2).

Table 2. Number of candidates retrieved from Adis Insight by condition.

|  | PTL/PTB | PE/E | IUGR | PPH | Fetal distress |
| --- | --- | --- | --- | --- | --- |
| Number of unique candidates identified via Adis Insight | 30^a^ | 14^a^ | 3^a^ | 12^b^ | 1^b^ |

^a^ Candidates identified through “drug” outputs.

^b^ Candidates identified through “clinical trial” outputs.

(b) We cross-checked candidates identified through Adis Insight with those from Citeline's Pharmaprojects^[[2]](#footnote-2)^ – a paid database output that offers end-to-end tracking of the global pharma R&D pipeline. The output was initially procured and retrieved by Concept Foundation in March 2020. Cross-check was performed in January 2021. All candidates identified in the Pharmaprojects search results were present in the Adis Insight results. Adis Insight returned additional relevant candidates not present in the Pharmaprojects output.

(c) We requested a data export from the World Health Organization (WHO) International Clinical Trials Registry Platform (ICTRP)^[[3]](#footnote-3)^ – the most comprehensive list of global clinical trials available. The following search terms were used to retrieve five datasets related to the project’s pregnancy-specific conditions in January 2021: “preterm labour” OR “preterm birth”; “pre-eclampsia” OR “preeclampsia”; “Impaired fetal growth” OR “IUGR” OR “fetal growth restriction”; “postpartum haemorrhage” OR “obstetric haemorrhage”; “fetal distress”. Clinical trials were scoped for relevance, which we defined as an investigation of one or more drugs, biologics, or dietary supplements with a primary and/or secondary outcome measure matching at least one of the five pregnancy-related conditions. This step served a dual function of uncovering additional candidates for inclusion that had not yet been identified (see Table 3), as well as capturing and linking clinical trial data to candidates marked for inclusion in the database (see Step 2 below for more information).

Table 3. Number of candidates retrieved from ICTRP by condition.

|  | PTL/PTB | PE/E | IUGR | PPH | Fetal distress |
| --- | --- | --- | --- | --- | --- |
| Number of unique clinical trials returned via ICTRP | 3,691 | 907 | 298 | 460 | 93 |
| Number of unique clinical trials identified as in scope | 363 | 304 | 72 | 267 | 4 |
| Number of unique candidates identified via in scope clinical trials | 41 | 60 | 25 | 27 | 2 |
| Number of additional candidates identified via ICTRP not already identified via Adis Insight | 32 | 57 | 24 | 17 | 1 |

(d) We searched PubMed^[[4]](#footnote-4)^ for relevant literature to validate already identified and uncover new candidates for inclusion. We anticipated this would include several candidates in preclinical development, and considered search terms that would return information on novel or innovative R&D. We searched using the same pregnancy-specific condition search terms used in our ICTRP search (see above), combined with the following additional terms: “prevention”; “treatment”; “innovation”; “discovery”; and “preclinical”. We included additional search terms to validate findings: drugs; medicine; and “biologics”. For each pregnancy-specific condition, we therefore performed the following searches:

- “*condition search term*” + “prevention” + “innovation”
- “*condition search term*” + “treatment” + “innovation”
- “*condition search term”* + “prevention” + “discovery”
- “*condition search term*” + “treatment” + “discovery”
- “*condition search term*” + “prevention” + “preclinical”
- “*condition search term*” + “treatment” + “preclinical”
- “*condition search term*” + “prevention” + drugs + medicine
- “*condition search term*” + “treatment” + drugs + medicine
- “*condition search term*” + “prevention” + “biologics”
- “*condition search term*” + “treatment” + “biologics”

PubMed searches were conducted between March and April 2021. Returned paper titles and abstracts were reviewed for relevance. Relevant publications were reviewed in full. Unique and in-scope candidates were added to the database, or additional data on existing candidates already entered in the database were captured (see Table 4).

Table 4. Number of publications (total/relevant^a^) retrieved, and candidates (additional) identified from PubMed by condition.

|  | PTL/PTB | PE/E | IUGR | PPH | Foetal distress |
| --- | --- | --- | --- | --- | --- |
| “*condition*” + “prevention” + “innovation” | 49/13 | 31/12 | 6/2 | 20/9 | 2/0 |
| “*condition*” + “treatment” + “innovation” | 32/6 | 25/9 | 8/2 | 21/3 | 3/0 |
| “*condition* + “prevention” + “discovery” | 34/11 | 0/0 | 3/0 | 1/1 | 0/0 |
| “condition” + “treatment” + “discovery” | 32/10 | 36/15 | 6/2 | 6/2 | 0/0 |
| “condition” + “prevention” + “preclinical” | 34/14 | 25/19 | 7/6 | 2/1 | 0/0 |
| “condition” + “treatment” + “preclinical” | 36/11 | 41/29 | 11/10 | 1/1 | 0/0 |
| “condition” + “prevention” + drugs + medicine | 104/60 | 84/44 | 14/7 | 51/42 | 2/2 |
| “*condition*” + “treatment” + drugs + medicine | 126/62 | 149/77 | 25/13 | 54/38 | 8/5 |
| “*condition*” + “prevention” + “biologics” | 2/1 | 3/2 | 0/0 | 0/0 | 0/0 |
| “*condition*” + “treatment” + “biologics” | 3/2 | 6/4 | 0/0 | 1/0 | 0/0 |
| Number of additional candidates identified via PubMed not already identified via ICTRP and Adis Insight | 116 | 80 | 35 | 10 | 9 |

^a^ Relevant papers are those in which we identified additional or validated existing candidates for any of the five conditions.

(e) We searched the grant databases of three of the largest global funders of medicines development to validate existing and find new candidates (particularly those in preclinical/discovery stage): the United States National Institutes of Health (US NIH)’s RePORTER^[[5]](#footnote-5)^; the European Union/Commission’s CORDIS^[[6]](#footnote-6)^; and the Bill & Melinda Gates Foundation (BMGF) grants database (data supplied from the BMGF). For all databases, we searched using the same pregnancy-specific condition search terms used for ICTRP (see above). For RePORTER, we retrieved all grants dating from 2000 to present. For CORDIS, we retrieved four datasets relating to different EC program cycles: FP5 1998–2002; FP6 2002–2006; FP7 2007–2013; and Horizon 2014–2020. For the BMGF, we searched datasets ranging from 2014–2019 inclusive (dates supplied and available for review). All datasets were retrieved and scoped for relevance between March and April 2021. Profiles were created for new candidates or information added to existing candidates, as appropriate (see Table 5).

Table 5. Number of grants and candidates retrieved from RePORTER, CORDIS, and the BMGF databases by condition.

|  | PTL/PTB | PE/E | IUGR | PPH | Fetal distress |
| --- | --- | --- | --- | --- | --- |
| **RePORTER** | | | | | |
| Number of grants returned via RePORTER | 587 | 198 | 152 | 31 | 65 |
| Number of grants identified as in-scope | 8 | 14 | 1 | 1 | 0 |
| Number of unique candidates identified via in-scope grants | 9 | 13 | 1 | 1 | 0 |
| Number of additional candidates identified via in-scope grants not already identified elsewhere | 0 | 2 | 1 | 0 | 0 |
| **CORDIS** | | | | | |
| Number of grants returned via CORDIS | 54 | 23 | 31 | 39 | 4 |
| Number of grants identified as in-scope | 0 | 0 | 0 | 0 | 0 |
| Number of unique candidates identified via in-scope grants | 0 | 0 | 0 | 0 | 0 |
| Number of additional candidates identified via in-scope grants not already identified elsewhere | 0 | 0 | 0 | 0 | 0 |
| **BMGF** | | | | | |
| Number of grants returned via database | 4 | 30 | 32 | 4 | 0 |
| Number of grants identified as in-scope | 2 | 0 | 0 | 3 | 0 |
| Number of unique candidates identified via in-scope grants | 1 | 0 | 0 | 1 | 0 |
| Number of additional candidates identified via in-scope grants not already identified elsewhere | 0 | 0 | 0 | 0 | 0 |

Step 2: Linking preclinical and clinical development data

For candidates in clinical development, we collected relevant clinical trial data through a few sources. Primary candidate identification through Adis Insight^[[7]](#footnote-7)^ (Step 1) also provided linked clinical trials. These were scoped for relevance, and manually uploaded to the clinical trial entries in our database. Next, we datamined the datasets retrieved from the WHO International Clinical Trials Registry Platform (ICTRP)^[[8]](#footnote-8)^ as described above. We scoped every clinical trial entry in the datasets for each pregnancy-specific condition. Relevant trials were marked for inclusion and assigned to a candidate (or multiple if more than one candidate was being investigated). Given the size (in the thousands), this data was then uploaded to the clinical trial entries in our database using a coded, automated upload. We cross-checked ICTRP clinical trials with those from Adis Insight to rule out duplicates. We also cross-checked candidates against relevant national clinical trials registers^[[9]](#footnote-9)^, if additional clinical trial information was needed. Clinical trial data scope and upload was performed between February and March 2021.

For candidates in preclinical development, results were sourced through PubMed searches between March and April 2021 (see Step 3).

Step 3: Completing candidate profiles

Much of the candidate information needed to complete candidate profiles was provided through Steps 1 and 2. The clinical trials served as a key source of information for completing candidate profiles, including information on route of administration, target, mode of action, and development status (among others). However, to ensure all fields were completed, we utilized academic literature search engines/tools to source greater detail and context for the candidates identified in Steps 1 and 2. Primarily, we searched PubMed^[[10]](#footnote-10)^ using the candidate name(s), and reviewed relevant literature retrieved (including that already sourced in Step 1) to verify and cross-reference information as needed. This type of information helped us to understand the development status/activity of candidates, as well as provided deeper information on those in preclinical development. Where possible, all references were standardized to PubMed URLs.

For candidates that failed or stagnated, additional information (on top of that provided by Adis Insight) was searched via relevant regulatory websites, such as the US Food and Drug Administration (FDA)^[[11]](#footnote-11)^ and European Medicines Agency (EMA)^[[12]](#footnote-12)^. Information for other database fields was sourced from a number of reliable online sources, including DRUGBANK Online^[[13]](#footnote-13)^, PubChem^[[14]](#footnote-14)^, the US National Library of Medicine’s Medical Subject Headings (MeSH) portal^[[15]](#footnote-15)^, and other websites as needed.

Additional candidate profile information was conducted between January and May 2021, concurrently with the steps outlined above.

Step 4: External validation and sense checking

Following database completion, a series of internal and external, independent reviews were undertaken to clean and validate the data. Internally, each candidate was reviewed for content, consistency, and logic by a minimum of two and often three or four individuals. Data cross-checking and cleaning was conducted in a rigorous, sequential manner. Some steps served to clean and standardize the data, while others were intended to identify content or subject matter error. Illustrative content checks included, for example, reviewing archetype against highest R&D stage (i.e. all “repurposed” candidates needed logically to have a highest R&D stage as “marketed”), or reviewing clinical use status by current R&D stage (i.e. all approved candidates needed logically to be Phase IV, with off-label or not yet approved candidates preclinical through to Phase III).

An external review was also undertaken. We sought independent, specialist input from two external reviewers actively working on new drugs development for one or more of the pregnancy-related conditions under investigation: Dr Sarah A. Marshall and Dr Neville Fields (Ritchie Centre, Department of Obstetrics and Gynecology, Monash University). The entire database was reviewed to validate candidates or identify known missing candidates; review the essential, standard labels for the candidates; review the fields related to clinical use case; and for each, recommend corrections, improvements, or additional details. Advisory input from the Concept Foundation team and the project’s expert advisory committee was also sourced throughout data compilation, as needed.

Key methodological decisions

As research unfolded and some new end-user requirements for the database were introduced, it was necessary to revisit, stress test, and occasionally make ongoing, minor adjustments or clarifications to the inclusion/exclusion criteria, as well as various database fields. Each change or refinement was made with the aim of maximizing standardization across the candidate profiles. These modifications were documented and include:

Methodological refinements related to inclusions/exclusions:

- Dietary supplements were broadly defined following the US FDA definition^[[16]](#footnote-16)^, but tailored for functionality and usability, for example to keep specific groupings of compounds together, such as polyphenols. Those specifically registered as a drug (such as N-acetyl cysteine) were kept as drugs. For inclusion, dietary supplements needed to be dosed formulations (i.e. food-based interventions such as “beetroot juice” or “chocolate” alone were not considered in scope).
- Candidates that addressed all stages of the pregnancy condition, including in some instances the postpartum period (e.g. postpartum pre-eclampsia) were included. However, candidates directed at postpartum conditions related to but not specifically targeted to the pregnancy-specific condition (e.g. cardiac disease in women who have had pre-eclampsia) were excluded.
- Antibiotics (e.g. azithromycin, erythromycin, clindamycin, amoxicillin, etc) investigated in or used for preterm labor (to prevent ascending infection and inflammation during premature rupture of membranes (PROM) or as a prophylactic in women with intact membranes) were excluded. This was agreed to based on their broad applicability well beyond that of the pregnancy-specific condition in question; the large number of them (with potential to skew the dataset); and their very downstream position in treating each condition’s pathology).
- Agents that inhibit uterine infection or inflammation (e.g. N-acetylcysteine, indomethacin, aspirin, progesterone, pravastatin, etc) or maintain vaginal flora/pH were included. This was decided due to their specificity to the specific inflammatory pathways identified as precursors to a number of the pregnancy-related conditions, and their upstream position in treating each condition’s pathology. Antenatal agents aimed at reducing consequences of preterm labor/birth (e.g. corticosteroids for fetal lung maturation) were included, given their frequent administration alongside tocolytics.
- Antihypertensives for pre-eclampsia/eclampsia (e.g. labetalol, methyldopa etc.) were included. Despite their broad applicability, they are critically used to prevent and treat pre-eclampsia/eclampsia, and as such are in scope.
- Broad hemostatic agents for treatment of general hemorrhage (e.g. prothrombin complex concentrate, fresh frozen plasma etc) were included only if PPH was specifically indicated for its use, or was investigated as an outcome.
- Candidates used only for experimental purposes (e.g. as an experimental aid to elicit smooth muscle contraction or relaxation) were only included if reference was made to/the research was geared towards one or more of the pregnancy-specific conditions.

Methodological refinements and clarifications related to data fields:

- *Archetype:* “Repurposed” candidates were any candidate previously or currently marketed for any other condition. “New chemical or biological entities (NCEs)” were candidates not already marketed for any condition (unless an NCE marketed for that pregnancy-related condition). We also considered candidates with new formulations or different routes of administration of existing medicines (e.g. inhalable oxytocin, heat stable carbetocin etc) as NCEs (unless already marketed for something else).
- *Clinical use status:* If verified in the literature, drug databases, FDA/EMA sites, or by one or more clinical practitioners/experts that a candidate was approved and marketed for clinical use, or is advised for or frequently used off-label for that condition in clinical practice, candidates were marked as such.
- *Current R&D stage:* Some candidates were in clinical development with no R&D stage specified in linked clinical trials, or stated as “N/A” or “unknown”. Others listed a variety of R&D stages amongst various clinical trials, including those marked as Phase IV even for candidates not yet marketed for that condition. To allocate a single, appropriate R&D stage to each candidate, we reviewed linked clinical trials against accepted definitions of each R&D stage (preclinical through to Phase IV), and assigned a phase based on trial descriptions.
- *Development status (active versus inactive):* Due to the often proprietary nature of R&D, information on the current development status of some candidates was not available in the public domain. To avoid labelling many candidates as development status “unknown”, it was agreed that active candidates would be any candidate with evidence of R&D within the last three years (since 2019). If no updates were made available on a candidate in the previous three years, or there was clear evidence of their discontinuation since then, they were marked as inactive.
- *Pharmacological subgroup:* This field was populated using either the WHO ATC 4th level classification for that drug or the FDC EPC classification.
- *Medical subject headings:* With numerous possible pharmacological and clinical classifications for medicines, as well as the great divergence in spelling protocols and naming conventions, the need for inclusion and standardization of this information became paramount. It was decided that this field would be populated first through a search for the candidate in the NIH – Medical Subject Headings (<https://meshb.nlm.nih.gov/search>) search engine. Items listed under the field labelled “Pharm Action” were included. If the candidate was not listed, any other useful keywords that described the pharmacological or therapeutic uses for the candidate were included (e.g. Bronchodilator agents; Sympathomimetics; Tocolytic agents etc).

Materials and platforms

Our database was built using Microsoft Lists and transposed to Microsoft Excel and Word. Our analyses were performed using Microsoft Excel.

Limitations

Our aim was to identify all medicines in development for these conditions since 2000, which we approached by utilizing the comprehensive, multipronged search strategy described above. However, due to the proprietary nature of (and lack of publicly available information on) many, particularly preclinical, candidate investigations, we anticipate the data may have gaps with respect to the full body of research. We also acknowledge that the data sources used that rely on self-reporting by investigators (e.g. ICTRP) have their own inherent limitations, including potential for reporting biases arising from changes in adherence and utilization over time. Lastly, readers should also note the data is up to date – and analyses drawn from the status of candidates – as of mid-2021.

1. <https://adisinsight.springer.com/> [↑](#footnote-ref-1)
2. <https://pharmaintelligence.informa.com/products-and-services/data-and-analysis/pharmaprojects> [↑](#footnote-ref-2)
3. <https://apps.who.int/trialsearch/> [↑](#footnote-ref-3)
4. <https://pubmed.ncbi.nlm.nih.gov/> [↑](#footnote-ref-4)
5. <https://reporter.nih.gov/> [↑](#footnote-ref-5)
6. <https://cordis.europa.eu/> [↑](#footnote-ref-6)
7. <https://adisinsight.springer.com/> [↑](#footnote-ref-7)
8. <https://apps.who.int/trialsearch/> [↑](#footnote-ref-8)
9. <https://sites.google.com/a/york.ac.uk/yhectrialsregisters/home/clinicaltrials> [↑](#footnote-ref-9)
10. <https://pubmed.ncbi.nlm.nih.gov/> [↑](#footnote-ref-10)
11. <https://www.fda.gov/home> [↑](#footnote-ref-11)
12. <https://www.ema.europa.eu/en> [↑](#footnote-ref-12)
13. <https://go.drugbank.com/> [↑](#footnote-ref-13)
14. <https://pubchem.ncbi.nlm.nih.gov/> [↑](#footnote-ref-14)
15. <https://meshb.nlm.nih.gov/search> [↑](#footnote-ref-15)
16. <https://www.fda.gov/food/information-consumers-using-dietary-supplements/questions-and-answers-dietary-supplements> [↑](#footnote-ref-16)
